# Molecular Phylogeography and Population Genetic Structure of *O. longilobus* and *O. taihangensis* (*Opisthopappus*) on the Taihang Mountains

**DOI:** 10.1371/journal.pone.0104773

**Published:** 2014-08-22

**Authors:** Yiling Wang, Guiqin Yan

**Affiliations:** College of Life Sciences, Shanxi Normal University, Linfen, China; St. Petersburg Pasteur Institute, Russian Federation

## Abstract

Historic events such as the uplift of mountains and climatic oscillations in the Quaternary periods greatly affected the evolution and modern distribution of the flora. We sequenced the *trn*L–*trn*F, *ndh*J-*trn*L and ITS from populations throughout the known distributions of *O. longilobus* and *O. taihangensis* to understand the evolutionary history and the divergence related to the past shifts of habitats in the Taihang Mountains regions. The results showed high genetic diversity and pronounced genetic differentiation among the populations of the two species with a significant phylogeographical pattern (*N*_ST_>*G*_ST_, *P*<0.05), which imply restricted gene flow among the populations and significant geographical or environmental isolation. Ten chloroplast DNA (cpDNA) and eighteen nucleus ribosome DNA (nrDNA) haplotypes were identified and clustered into two lineages. Two corresponding refuge areas were revealed across the entire distribution ranges of *O. longilobus* and at least three refuge areas for *O. taihangensis*. *O. longilobus* underwent an evolutionary historical process of long-distance dispersal and colonization, whereas *O. taihangensis* underwent a population expansion before the main uplift of Taihang Mountains. The differentiation time between *O. longilobus* and *O. taihangensis* is estimated to have occurred at the early Pleistocene. Physiographic complexity and paleovegetation transition of Taihang Mountains mainly shaped the specific formation and effected the present distribution of these two species. The results therefore support the inference that Quaternary refugial isolation promoted allopatric speciation in Taihang Mountains. This may help to explain the existence of high diversity and endemism of plant species in central/northern China.

## Introduction

During the last three million years, repeated glacial and interglacial cycles have greatly affected the landform and fauna of the total earth [1]–[3]. Historical ecological and biogeographic factors are often considered to have played significant roles in shaping global biodiversity by influencing regional differences in speciation, extinction, and migration [4]–[6]. The genetic structures of the current species record the simultaneous consequences of two fundamental processes: population dynamics in response to past geological or climatic changes, and lineage sorting within a species under natural selection. Thus, knowledge of the evolutionary history of many plant species is central to the identification of divergence and speciation processes [3], [7]–[8].

Phylogeographical analyses can provide an understanding of how paleo-environmental changes in landscape and climate have influenced species distributions and population demography [9]. Such analyses play an important role in understanding the evolutionary history of species with changes [3]. Over the last decade, molecular research on the evolutionary history of plant species, both in China and throughout East Asia, has been conducted with reference to past climatic oscillations [10]–[18]. One of the results of such research is the proposal by some authors that glacial refugia were maintained in both the northern and the southern regions, or at different spatial-temporal scales in China, during these glacial periods. These refugia are thus suggested to have acted as sites for subsequent range expansion during the interglacial (or postglacial) periods [12]–[14], [19]–[20]. Some plant species have extended their distribution from southwestern China to central/northern China [21]. Reduction of genetic diversity is expected under a scenario of rapid postglacial expansion, as has been found in northern Europe and America [2]. Because central/northern China is believed not to have been glaciated during the Quaternary [2], [22]–[23], it is an open question whether species experienced past northern expansion and southern retreat during the Quaternary climatic oscillations.

Taihang Mountains locates on 35°19′–40°51′N and 113°10′–115°48′E. As a natural boundary mountain of the east, southeast of Shanxi province and Hebei and Henan provinces, it is the important mountain range and geographical boundary for eastern China. The northern part of Taihang Mountains is higher than its southern part. Most areas of Taihang Mountains are higher than 1200 meters. The uplift of Taihang Mountains, followed by the formation of high mountains and deep valleys within the plateau [24]–[25], was one of the most important geological events. Several lines of evidence suggest that the rapid uplift of Taihang Mountains took place after the late Pleistocene [26]–[27]. Rich sources of species, most of which are endemic, are found in this region [24]. The high species richness in Taihang Mountains has led to the hypothesis that this region is a distribution and diversity center for many plant genera [24].

Molecular techniques have provided powerful tools for studying the phylogeography or migratory footprints of species [7]–[9], [28]–[29]. Maternally inherited chloroplast DNA lineages in natural populations often display geographical structure [9], which is useful for deciphering the evolutionary history of species. The cpDNA markers are thought to be the most appropriate candidates because of their slow evolution and lack of recombination [30]. Nevertheless, the joint use of molecular markers derived from different genomes provides a more complete description of population structure and insights into population history and dynamics, particularly for comparisons of maternally inherited organelle and bi-parentally inherited nuclear markers [31]. Several studies about inter-specific and intra-specific divergence from China, using different molecular markers, have played an important role in discovering phylogeographical patterns in East Asian flora. This is particularly true for the mechanisms of plant speciation, along with the production of the high plant biodiversity and endemism found in East Asian flora [12]–[13], [15], [20], [32]. However, most of the studied species from China have been trees [13], [33]–[34]. Herbaceous vegetation has received much less attention [16]. Therefore, it would be of great interest to study the phylogeography of an herbaceous species to understand the evolution and modern distribution of the vegetation, especially in Taihang Mountains of central/northern China.

*Opisthopappus longilobus* Shih and *Opisthopappus taihangensis* (Ling) Shih belong to *Opisthopappus* (Asteraceae). The genus *Opisthopappus* is endemic to China, and its wild distribution is mainly restricted to Taihang Mountains across the provinces of Shanxi, Hebei, and Henan, occurring on the slopes at an elevation of about 1000 m or in the cracks of the steep cliffs [35]. *O. longilobus* is mainly distributed in the province of Hebei, whereas *O. taihangensis* is found in the provinces of Shanxi and Henan [35]–[36]. Both species are diploid (2n = 18) [25]. A strict morphological differentiation occurred between *O. longilobus* and *O. taihangensis*. The leaf blade of *O. longilobus* is smooth and subcylindrical, and most stem and leaves are pinnatifid, with one pair of bracteal leaf. *O. taihangensis*, apprised pubescent on both surfaces of leaf blade, stem, and leaves, is bipinnatifid, with no bracteal leaf [35]–[36]. *O. longilobus* and *O. taihangensis* have been listed among the Class II State-Protected Endangered Plant Species [36] due to the narrow distribution range, unique ecological environment, and serious artificial plucking.

*O. longilobus* and *O. taihangensis* are an ideal candidate for investigating the influences of Quaternary climate change in Taihang Mountains for the following reasons: (i) Geographically, *O. longilobus* and *O. taihangensis* are distributed only in Taihang Mountains, including Shanxi, Hebei, and Henan provinces. This geographic range may be advantageous for enabling investigation into whether the glacial refuges were maintained in Taihang Mountains during the last glacial maximum, or earlier cold periods. (ii) *O. longilobus* and *O. taihangensis* have intra-specific and endemic taxa in China that display visible phenotypic differences, which are important in exploring the incipient speciation caused by isolation provided by a glacial refuge. Finally, (iii) *O. longilobus* and *O. taihangensis* are an herbaceous plant and sensitive to environmental changes. In research on the effects of climate oscillations on plant evolution, it can represent the phylogeography of an herb, and can serve as a model to reveal the historical and evolutionary processes of such plants since the period of Quaternary climate change.

Thus, our objectives included the following: (1) explore the phylogeographical structure and the evolutionary history of *O. longilobus* and *O. taihangensis*; (2) elucidate the effects of the uplift of Taihang Mountains and the Quaternary climatic oscillations on the genetic structure, divergence and distribution of *O. longilobus* and *O. taihangensis*; (3) infer the possible refugia of *O. longilobus* and *O. taihangensis* across their whole distribution range.

## Materials and Methods

### Ethics statement

This study was conducted in accordance with all People's Republic of China laws. No specific permits were required for the described field studies because all researchers collecting the samples had introduction letters from the College of Life Science, Shanxi Normal University, Linfen.

### Plant sampling

**Figure 1:**
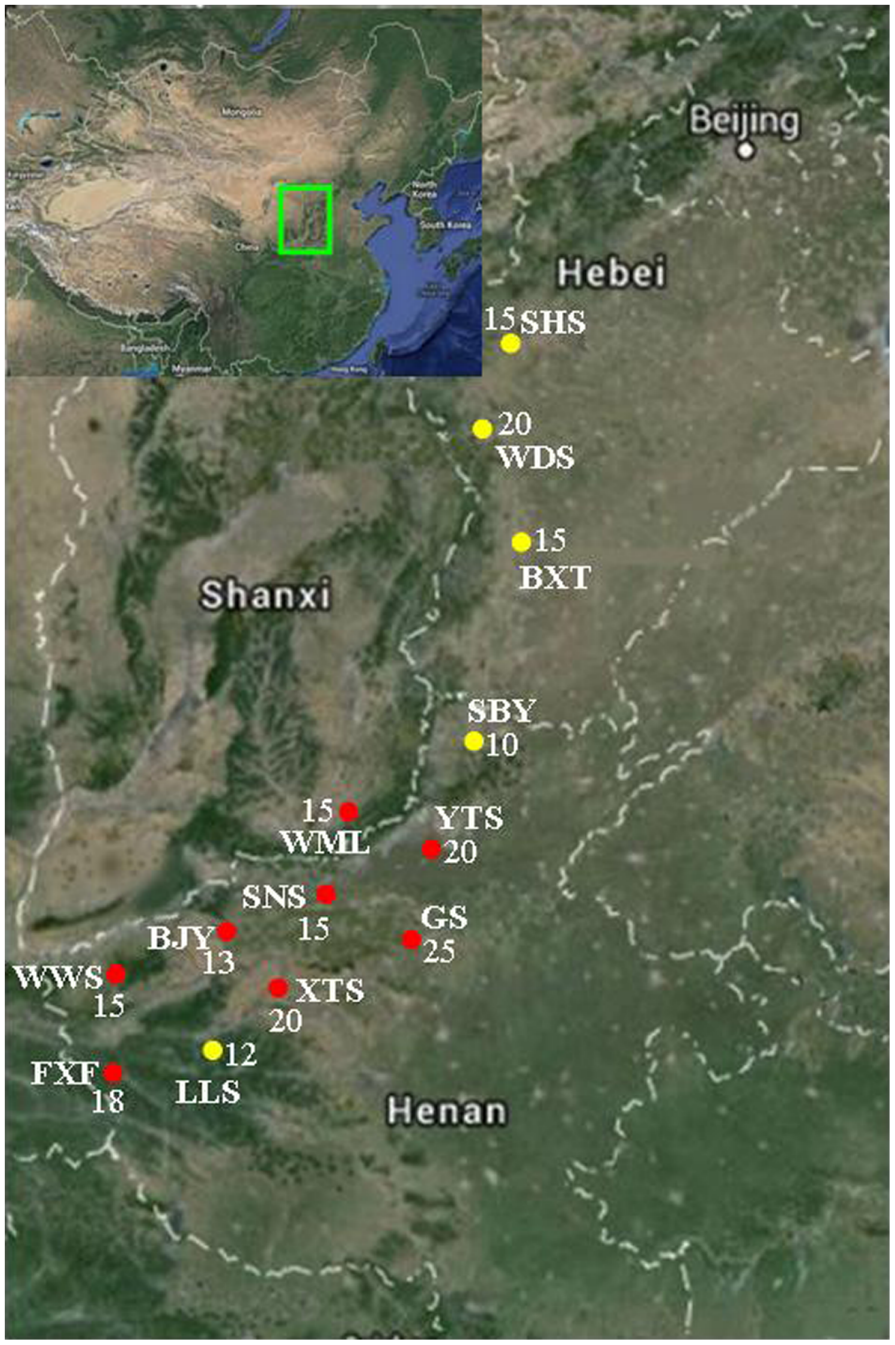
Map of sample sites for *Opisthopappus* (Asteraceae) on the Taihang Mountains. Location details are given in Table 1, and locality numbers correspond to those in Table 1. Yellow circle dots represent the populations of *O. longilobus*; red circle dots represent the populations of *O. taihangensis*.

Thirteen populations of *Opisthopappus* were investigated in this study, covering the entire distribution area on Taihang Mountains. The samples comprised five populations of *O. longilobus* and eight populations of *O. taihangensis* (Table 1, Figure 1). Each population included 10 to 25 individuals that were collected at least 5 m apart. Healthy leaves were collected and dried in silica gel until total DNA was extracted. In each of the 13 populations, the ecological factors of each location were determined and recorded (Table 1).

**Table 1:** Location of the sampled *Opisthopappus* populations and the estimated diversity indexes.

Population		Geographic origin	Sample size	Latitude	Longitude	Number hapelotypes cpDNA (ITS)	Haplotype diversity cpDNA (ITS)	Nucleotide diversity cpDNA (ITS)
*O. longilobus*	WDS	Wudangshan, Hebei	20	113°47′	36°57′	6(7)	0.854(0.819)	0.00194(0.00240)
	BXT	Beixiangtang, Hebei	15	114°09′	36°02′	1(3)	0.000(0.556)	0.00000(0.00130)
	SHS	Shuanghuangshan, Hebei	15	113°57′	36°54′	2(3)	0.500(0.833)	0.00119(0.00391)
	LLS	Linlvshan, Henan	12	113°02′	34°43′	1(5)	0.000(0.857)	0.00000(0.00196)
	SBY	Shibanya, Henan	10	113°32′	36°03′	1(1)	0.000(0.000)	0.00000(0.00000)
Species level						8(15)	0.838(0.908)	0.00175(0.00537)
*O.taihangensis*	GS	Guanshan, Henan	25	113°34′	34°45′	1(3)	0.000(0.714)	0.00000(0.00251)
	YTS	Yuntaishan, Henan	20	113°25′	35°25′	2(2)	0.500(0.400)	0.00089(0.00059)
	XTS	Xiantaishan, Henan	20	113°49′	34°11′	2(1)	0.600(0.000)	0.00107(0.00000)
	SNS	Shennongshan, Henan	15	112°44′	35°16′	1(3)	0.000(0.618)	0.00000(0.00155)
	FXF	Foxifeng, Henan	18	112°62′	35°11′	1(3)	0.000(0.607)	0.00000(0.00162)
	WWS	Wangwushan, Henan	15	112°26′	35°38′	1(3)	0.000(0.700)	0.00000(0.00176)
	BJY	Baijiayan, Henan	13	112°92′	35°46′	2(2)	0.667(0.667)	0.00040(0.00293)
	WML	Wangmangling, Shanxi	15	113°35′	35°41′	2(2)	0.533(0.667)	0.00063(0.00098)
Species level						5(6)	0.562(0.558)	0.00073(0.00158)

### DNA extraction, amplification and sequencing

Total DNA was extracted from the silica gel–dried leaves using the modified 2×CTAB procedure [37]. DNA quality was checked by electrophoresis on 0.8% agarose gels, and DNA concentration was determined using an Eppendorf biophotometer protein nucleotide analyzer (Eppendorf China Ltd., Beijing, Germany). The DNA samples were diluted to 10 ng·µL^−1^ and stored at 20°C for subsequent use.

The *trn*L-*trn*F sequences were amplified using *trn*L and *trn*F [38].The primers *ndh*J and *trn*L [39] were used to amplify the *ndh*J-*trn*L sequences. ITS sequences were then amplified using the primers ITS4 and ITS5 [40]. A polymerase chain reaction (PCR) was then performed in a 25 µL volume, with 50 ng plant DNA, 2×MasterMix (0.2 mM dNTPs, 3 mM MgCl_2_, 1×PCR buffer, and 0.1 unit Taq DNA polymerase), and 0.6 mM of both forward and reverse primers. The PCR parameters for all amplification programs of ITS and cpDNA were as follows: 4 min of pre-denaturation at 94°C, followed by 34 cycles of 30 s of denaturation, 40 s of annealing (49.2°C for ITS or 58.8°C for *trn*L-*trn*F or 59.8°C *ndh*J–*trn*L), 1 min 20 s of elongation at 72°C, and a final elongation step of 7 min at 72°C. PCR products were purified using the Wizard PCR Preps DNA purification system (Promega, Madison, WI, USA) following the manufacturer's instructions. Cycle sequencing reactions were conducted using the purified PCR product, AmpliTaq DNA polymerase, and fluorescent BigDye terminators. The sequencing products were analyzed using an ABI Prism 310 DNA sequencer (Applied Biosystems Inc., Foster City, CA, USA).

### Data analysis

The sequences were edited manually based on the chromatograms and aligned by CLUSTAL X [41] and then adjusted manually. Inserts and indels within all cpDNA and nrDNA sequences were firstly treated as a single character resulting from one mutation. Haplotype diversity (*h*) and nucleotide diversity (π) were calculated for each population (*h*, *π*) and at the species level (*h*_d_, *π*_d_) using DNAsp [42]. The program PERMUT [44] was used to calculate the within-population diversity (*h*_S_), total diversity (*h*_T_), geographical total haplotype diversity (*V*_T_), geographical average haplotype diversity (*V*_S_), level of population differentiation at the species level (*G*_ST_), and an estimate of population subdivisions for phylogenetically ordered alleles (*N*_ST_). We further tested the phylogeographical structure at the species range between (*N*_ST_) and (*G*_ST_) by using the U-statistic. An *N*_ST_ value higher than the *G*_ST_ value indicates that closely related haplotypes are observed more often in a given geographical area than would be expected by chance [43].

To quantify the genetic differentiation partitioned among different groups and total genetic variance, analyses of molecular variance (AMOVA) were carried out using ARLEQUIN [44], with 1000 random permutations to test for significance of partitions. The spatial genetic structure of haplotypes was analyzed by spatial analysis of molecular variance using SAMOVA [45]. This program uses a simulated annealing approach to define groups of populations (*K*) that are geographically homogenous and maximally differentiated from each other. In this analysis of haplotypes, *K* varied from 2 to 13, with each simulation starting from 100 random initial conditions. An *F*_CT_ index of genetic differentiation among initial *K* groups was computed, followed by an iterative simulated annealing process to obtain the optimal configuration of groups and final *F*_CT_ values. The simulated annealing process was repeated 1000 times. The configuration with the largest *F*_CT_ value among the 100 tested was retained as the best grouping of populations.

Genealogical relationships between haplotypes were inferred from a maximum parsimony median-joining network calculated in NETWORK 4.5.0.2 [46]. To complement the results of NETWORK, TCS 1.21 [47] was also used to construct haplotype relationships. Phylogenetic analyses were conducted for the nrDNA and cpDNA sequence data using maximum likelihood (ML) and Bayesian inference (BI), respectively.

The time of the most recent common ancestor (TMRCA) of all haplotypes was estimated via a Bayesian approach implemented in *BEAST (Star BEAST) [48]–[49] using a GTR+I substitution model. The best substitution model was determined according to the Akaike Information Criterion (AIC) in jModeltest [50]. These models were applied in ML, BI, and subsequent *BEAST analyses. Bayesian inference was performed using MrBayes 3.1.2 [51]. Two independent runs of Metropolis-coupled Markov chain (MCMC) analysis were executed, each including one cold chain and three incrementally heated chains that started randomly in the parameter space. Two independent runs of 10^8^ generations were carried out, with sampling at every 1000 generations. The first 25% of sampled trees were discarded as burn-in, and the remaining trees were used to construct a Bayesian consensus tree. The convergence of chains was checked using Tracer 1.5 [52]. The remaining trees were pooled to estimate the posterior probabilities (PPs).

No fossil records or biogeographic events isolating distinct populations are available to calibrate a cpDNA-IGS substitution rate for *O. longilobus* and *O. taihangensis*. Therefore, for our combined chloroplast non-coding regions, we assumed minimum and maximum values of a range of average mutation rates reported for synonymous sites of plant chloroplast genes [i.e., 1.2 and 1.7×10^−9^ substitutions per site per year (s/s/y)] [53]. These rates were then used for estimating TMCRA in *BEAST under a relaxed molecular clock assumption.

To detect whether population groups experienced recent population expansion and satisfied the assumption of neutrality, a mismatch distribution analysis was performed using the DNAsp program. Tajima's D and Fu and Li's D* were calculated for the entire genus and groups of populations. The statistical significance of D and D* was estimated with coalescent simulations as implemented in this program [54]–[55]. To further infer demographic processes, the null hypotheses of a spatial expansion and of a pure demographic expansion were tested in ARLEQUIN by comparing observed and expected distributions of pairwise sequence differences (mismatch distributions). For each expansion model, goodness-of-fit was tested using the sum of squared differences (*SSD*) and Harpending's [56] raggedness index (HRag).

Finally, isolation by distance (IBD) for both nrDNA and cpDNA data was tested based on pairwise geographical and genetic distances (*F*_ST_) with the Isolation by Distance Web Service [57] (http://ibdws.sdsu.edu/~ibdws/), running 10,000 Mantel permutations.

## Results

### Patterns of variability in cpDNA and ITS sequences

**Figure 2:**
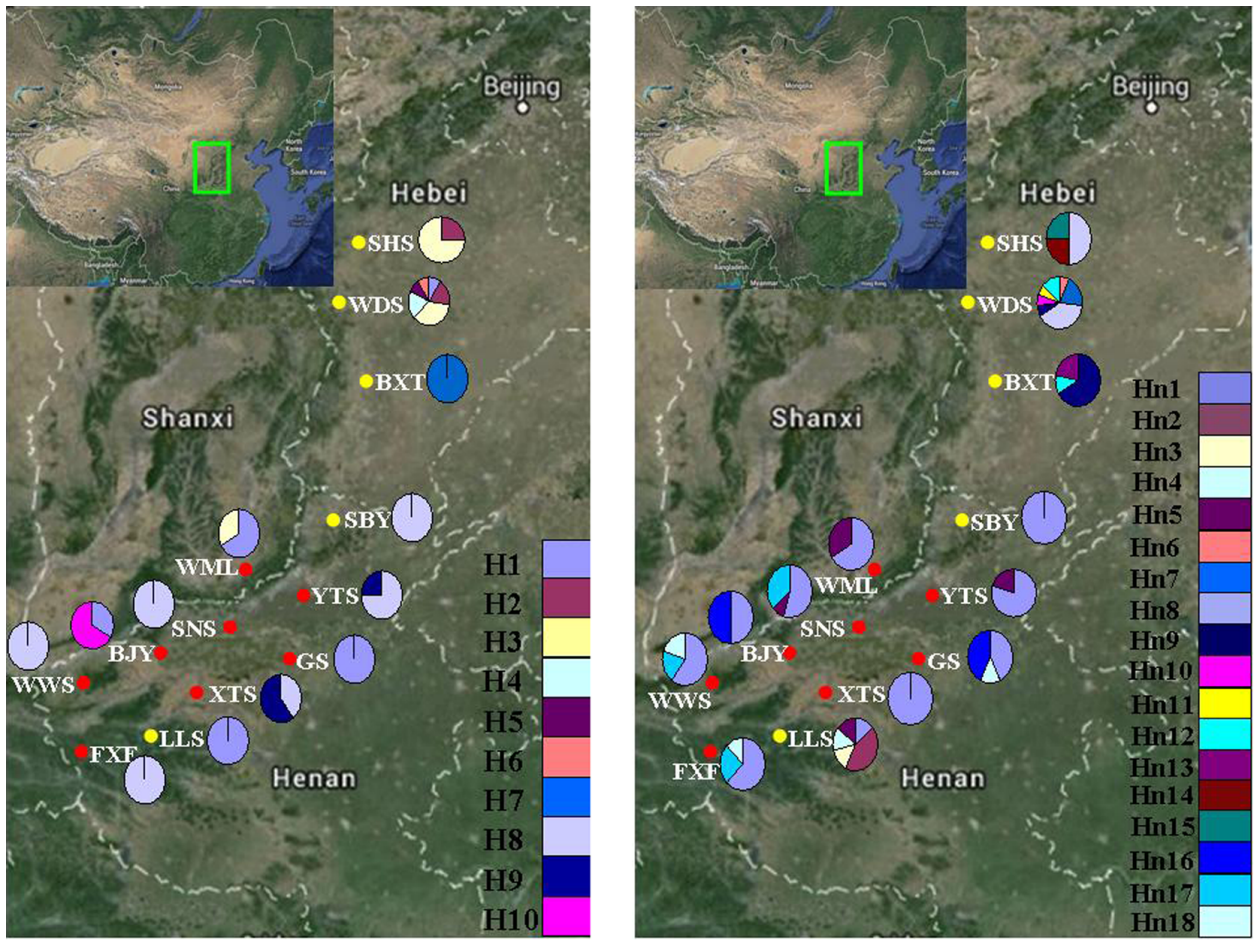
Geographical distribution of 10 cpDNA (A) and 18 (B) nrDNA haplotypes. The pie charts reflect the frequency of haplotype occurrence in each population. Haplotype colours correspond to those shown in panel.

The *trn*L-*trn*F and *ndh*J-*trn*L intergenic spacers varied from 800 to 866 bp and from 790 to 835 bp, respectively. The *trn*L-*trn*F intergenic spacer, which exhibits considerable nucleotide polymorphism, is characterized by 10 substitutions and a 4 bp insertions and deletions (Table S1). The 4 bp insertions and deletions region was mainly observed in *O. longilobus* populations. The *ndh*J-*trn*L spacer was characterized by one indel, three substitutions, and a 10 bp insertions and deletions (Table S1). The total cpDNA combined matrix comprised 1701 sites, of which 13 positions were variable and 18 harbored gaps. Ten haplotypes (Figure 2A) were observed by combining cpDNA data: eight haplotypes were within *O. longilobus* populations and five were within *O. taihangensis* populations. The haplotype diversity (*h*) ranged from 0 to 0.854, and the nucleotide diversity (*π*) from 0 to 0.00194 in the 5 populations of *O. longilobus* (Table 1). At species level, *h*_d_ = 0.838 and *π*_d_ = 0.00175 for *O. longilobus*. Within 8 *O. taihangensis* populations, *h* ranged from 0 to 0.667, *π* from 0 to 0.00107, *h*_d_ was 0.562 and *π*_d_ was 0.00073. The highest haplotype diversity was observed in population WDS from *O. longilobus* and BJY from *O. taihangensis* (Table 1).

The full ITS sequences, including ITS1+5.8S+ITS2, were 682 bp in length, comprising of 254 bp and 224 bp for ITS1 and ITS2, respectively. Eighteen haplotypes (Figure 2B) and 17 polymorphic sites, the latter consisting of 15 parsimony informative sites and 2 singleton variable sites, were detected at ITS sequences. Two populations, SBY and XTS, were monomorphic, whereas the remaining populations were polymorphic (Table 1). The haplotype diversity (*h*) ranged from 0 to 0.857 and the nucleotide diversity (*π*) from 0 to 0.00391 in the 5 populations *O. longilobus*. Within 8 *O. taihangensis* populations, the haplotype diversity *h* ranged from 0 to 0.714 and the nucleotide diversity *π* from 0 to 0.00293. For *O. longilobus*, *h*_d_ = 0.908, *π*_d_ = 0.00537; for *O. taihangensis* populations, *h*_d_ = 0.558, *π*_d_ = 0.00158 (Table 1).

### Genetic diversity and population structure

A genetic diversity analysis revealed high genetic diversity harbored in *O. longilobus* and *O. taihangensis* populations. The parameters *h*_T_, *V*_T_, *h*_S_, and *V*_S_ were 0.908, 0.916, 0.669, and 0.581, respectively, for *O. longilobus* based on cpDNA sequences. By ITS sequences, *h*_T_ = 0.944, *V*_T_ = 0.937, *h*_S_ = 0.824, and *V*_S_ = 0.889 within *O. longilobus* populations. For *O. taihangensis* populations, *h*_T_ = 0.828, *V*_T_ = 0.833, *h*_S_ = 0.531, and *V*_S_ = 0.455 by cpDNA sequences; *h*_T_ = 0.806, *V*_T_ = 0.810, *h*_S_ = 0.704, and *V*_S_ = 0.592 with nrDNA sequences.

Based on ITS sequences, phylogenetic analyses were carried (Figure S1). All studied populations made up a single monophyletic lineage, indicating that all of these divergent populations were derived within *Opisthopappus* itself, rather than the result of introgression by other species. Spatial genetic analyses of cpDNA and nrDNA haplotypes in all 13 populations using SAMOVA indicated that *F*_CT_ increased to a maximal value of 0.921 when *K* = 2 (*K*, the number of groups). Thus, the division of all the 13 sampled populations approximately into two groups is appropriate.

AMOVA analysis revealed that 60.58% and 67.70% (*P*<0.01) of the total variation was due to differences among the cpDNA and nrDNA populations for all studied populations, respectively (Table 2). When the populations were grouped by taxonomic variety, the cpDNA showed 27.28% variation among populations within species (*P*<0.01; Table 2). Similarly, for the nrDNA, AMOVA revealed that 26.68% of the variation was attributed to differences among populations within the species (*P*<0.01; Table 2). There was a significant (*P*<0.01) variation between species, suggesting that the two separately distributed groups have a genetic differentiation. In addition, 60.71% of the total cpDNA variation and 68.24% of the total nrDNA variation existed among *O. longilobus* populations (Table 2). The genetic structure of *O. taihangensis* populations showed a similar trend with that of *O. longilobus* populations, with most of the genetic differentiation occurring among populations (Table 2).

**Table 2:** Analyses of Molecular Variance (AMOVA) based on the nrDNA (ITS) and cpDNA sequences.

Source of variation	d.f	Sum of squares	Variance components	Variation percentage	Fixation indices
ITS(all population)					
Among populations	19	105.101	1.218	67.70	*F*_ST_ = 0.677 (*P*<0.01)
Within populations	178	44.179	0.581	32.30	
ITS (species)					
Among species	1	54.511	1.113	48.16	*F*_CT_ = 0.482(*P*<0.01)
Among populations within species	18	50.590	0.617	26.68	*F*_ST_ = 0.748(*P*<0.01)
Within populations	178	44.179	0.581	25.16	*F*_SC_ = 0.515(*P*<0.01)
ITS(*O. longilobus*)					
Among populations	12	44.767	1.499	68.24	*F*_ST_ = 0.682 (*P*<0.01)
Within populations	76	23.022	0.698	31.76	
ITS(*O. taihangensis*)					
Among populations	14	21.157	0.492	90.04	*F*_ST_ = 0.900 (*P*<0.01)
Within populations	95	5.823	0.054	9.96	
ITS (SMOVA species)					
Among species	7	57.023	2.456	50.21	*F*_CT_ = 0.502(*P*<0.01)
Among populations within species	12	55.147	2.078	28.34	*F*_ST_ = 0.786(*P*<0.01)
Within populations	178	40.563	1.789	21.45	*F*_SC_ = 0.497(*P*<0.01)
cpDNA(all population)					
Among populations	19	78.452	2.187	60.58	*F*_ST_ = 0.606 (*P*<0.01)
Within populations	178	50.234	1.023	39.42	
cpDNA (species)					
Among species	1	60.231	1.586	46.59	*F*_CT_ = 0.466(*P*<0.01)
Among populations within species	18	58.427	0.789	27.28	*F*_ST_ = 0.738(*P*<0.01)
Within populations	178	45.126	0.567	26.13	*F*_SC_ = 0.533(*P*<0.01)
cpDNA (*O. longilobus*)					
Among populations	12	29.313	1.032	60.71	*F*_ST_ = 0.607 (*P*<0.01)
Within populations	76	19.364	0.668	39.29	
cpDNA (*O. taihangensis*)					
Among populations	14	21.601	0.467	68.64	*F*_ST_ = 0.686 (*P*<0.01)
Within populations	95	9.183	0.214	31.36	
cpDNA (SMOVA species)					
Among species	7	56.273	1.756	52.34	*F*_CT_ = 0.523(*P*<0.01)
Among populations within species	12	55.012	1.111	27.55	*F*_ST_ = 0.799(*P*<0.01)
Within populations	178	41.603	0.654	22.36	*F*_SC_ = 0.498(*P*<0.01)

Results of the Mantel test showed a significant correlation between nrDNA data differentiation among populations and the natural logarithm of the geographical distances throughout the sampled range of *O. longilobus* (*r* = 0.442; *P* = 0.001) and *O. taihangensis* (*r* = 0.385; *P* = 0.001). Likewise, a significant correlation was detected among populations from cpDNA data of *O. longilobus* (*r* = 0.401; *P* = 0.001) and *O. taihangensis* (*r* = 0.399; *P* = 0.001). This indicates a significant correlation between genetic differentiation and geographical distance, implying strong IBD.

### Haplotype and phylogeographical structure

**Figure 3:**
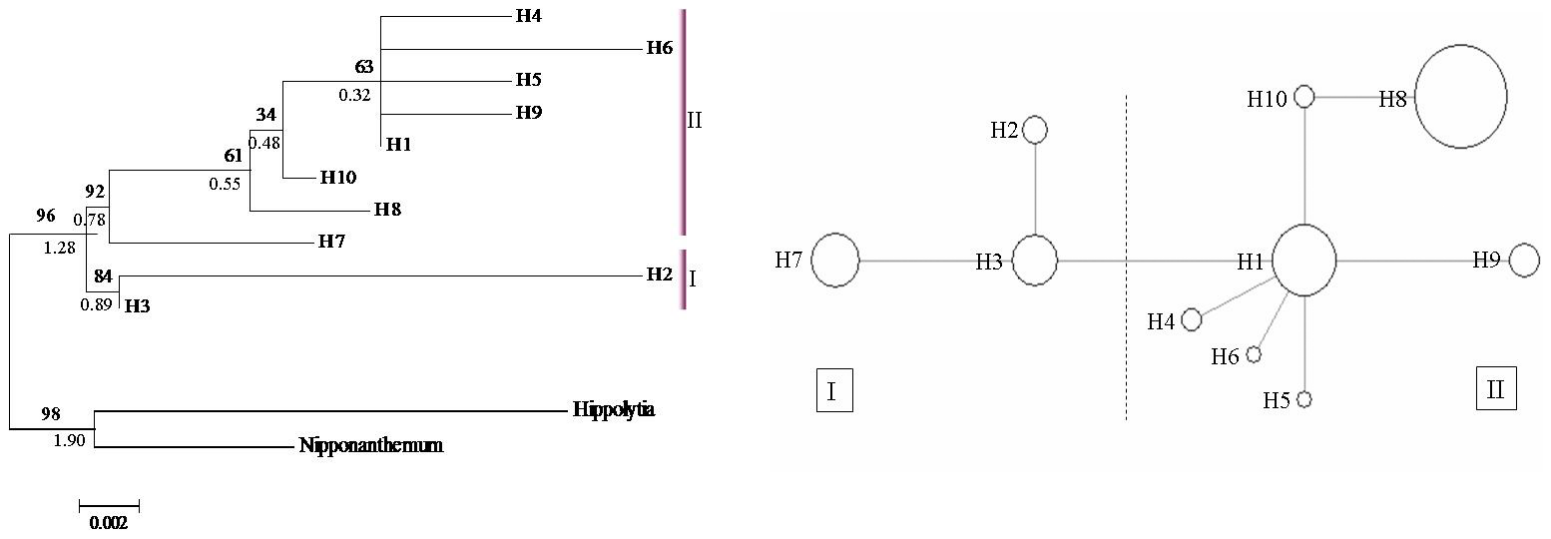
The evolutionary relationships among cpDNA haplotypes. (A) NJ phylogenetic tree for the 10 cpDNA haplotypes. Numbers above branches are support values from bootstrap resampling/Bayesian inference. (B) Median-joining network. Sizes of the circles are proportional to the overall frequency of the haplotypes in the entire sample of all species.

**Figure 4:**
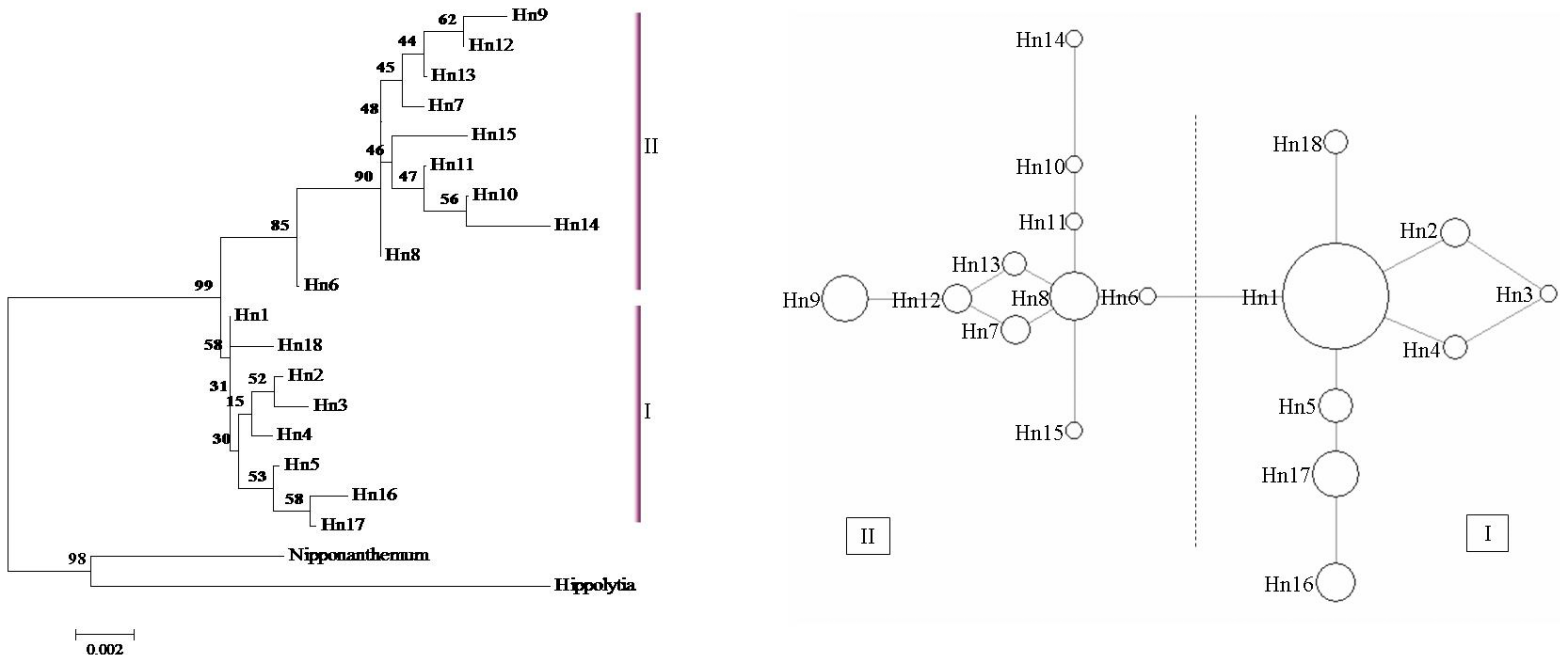
The evolutionary relationships among nrDNA haplotypes. (A) NJ phylogenetic tree for the 18 nrDNA haplotypes. Numbers above branches are support values from bootstrap resampling. (B) Median-joining network. Sizes of the circles are proportional to the overall frequency of the haplotypes in the entire sample of all species.

In this study, similar topologies were obtained from the two different network approaches used to infer the relationships among the cpDNA and nrDNA haplotypes. Only the median-joining network is shown. The haplotype H8 was the most frequent haplotype in cpDNA data, occurring in 6 of the 13 populations. Three haplotypes, H1, H3, and H8, were shared by *O. longilobus* and *O. taihangensis* populations (Table 1 and Figure 3). In nrDNA data, Hn1 was the most frequent haplotype, occurring in 10 of the 13 populations (Figure 3 and Figure 4). The haplotypes Hn1, Hn4, and Hn5 were shared by *O. longilobus* and *O. taihangensis* populations (Table 1 and Figure 4). The distributions of haplotypes that were restricted to a single population were extremely skewed. Four cpDNA and nine nrDNA haplotypes were found in *O. longilobus* single populations. Only one cpDNA haplotype was identified for *O. taihangensis* populations.

Based on cpDNA data, we estimated a *G*_ST_ value (0.649, *P*<0.05), which is significantly smaller than the *N*_ST_ value (0.761). When the nrDNA was examined, the *G*_ST_ value (0.253) also differed significantly from the *N*_ST_ value (0.641). A significant phylogeographic structure was indicated by both cpDNA and nrDNA data (*N*_ST_>*G*_ST_, *P*<0.05).

The phylogeographical relationships of the 10 cpDNA haplotypes and 18 nrDNA haplotypes were assessed under Neighbor-joining analyses (NJ) and Bayesian inferences drawn using *Hippolytia* and *Nipponanthemum* as outgroups (Figure 3 and Figure 4). Similar topology to phylogeographical relationships indicated a single phylogenetic split. All haplotypes were clustered into two lineages: I and II (Figure 3 and Figure 4). Lineage I contained some populations of *O. longilobus* with a high bootstrap support. The remaining haplotypes formed lineage II, containing some *O. longilobus* populations and all *O. taihangensis* populations, also with a high bootstrap support. This unrooted network of cpDNA haplotypes is consistent with the strict consensus tree produced by NJ and Bayesian inference and furthermore displays the relationship of interior (ancestral) and tip (derived) haplotypes (Figure 3). For lineage I (Figure 3), the haplotype H3 located in the center and fixed in three populations, being an ancestral haplotype. In clade II, H1 was shared by five populations. It appears to be an ancestral haplotype distributed in the center of clade II. The haplotype H8 was located in the tip of the network despite though contained a higher frequency of haplotypes. In the nrDNA haplotype network (Figure 4), Hn8 was ancestral for the lineage I; Hn1 was the predominant and widespread one in the clade II.

To test whether the current expansion had occurred in different populations, we calculated the frequency distribution of pairwise nucleotide differences among *O. longilobus* populations and *O. taihangensis* populations respectively. The resultant mismatch distribution consisted of multiple multimodal curves (Figure S2). Neutrality tests revealed nonsignificant positive values in considering all populations (Table 3). The observed mismatch distributions of cpDNA haplotypes were multimodal for all groups except for *O. taihangensis*. The mismatch distributions based on nrDNA haplotypes were present a similar pattern with cpDNA data (Table 3). Thus, a recent population expansion for *O. longilobus* is unlikely. *O. taihangensis* perfectly fits the expected expansion distribution (Figure 3).

**Table 3:** Parameters of mismatch distribution analysis.

	Tajima's D	*P*	Fu's Fs	*P*	Fu and Li's D*	*P*	Fu and Li's F*	*P*	*SSD*	*P*	*H*_RAG_	*P*
ITS data												
*O. longilobus*	0.085	>0.10	−3.988	0.012	0.283	>0.10	0.257	>0.10	0.111	<0.05	0.461	<0.05
*O. taihangensis*	0.483	>0.10	−0.787	0.157	1.002	>0.10	0.984	>0.10	0.106	<0.05	0.350	<0.05
cpDNA data												
*O. longilobus*	0.017	>0.10	0.271	0.182	0.012	>0.10	0.015	>0.10	0.168	<0.05	0.424	<0.05
*O. taihangensis*	0.261	>0.10	0.495	0.217	1.095	>0.10	0.977	>0.10	0.254	<0.05	0.806	<0.05

Divergence times were estimated using the program *BEAST. The *ESS* values were from 150 to 240 for all the nodes discussed as follows. All sampled haplotypes of *Opisthopappus* coalesced at about 1.90 Ma (95% highest posterior density, HPD) (0.50–1.72), which indicated the origin of *Opisthopappus* during the late Tertiary periods. The divergence times, estimated at approximately 1.28 Ma (95% HPD) (0.70–2.34) between *O. longilobus* and *O. taihangensis*, are shown in the haplotype phylogenic tree (Figure 3A). *O. longilobus* and *O. taihangensis* thus appear to have diverged from each other during the early Pleistocene.

## Discussion

### Taxonomic implications

At the morphological character levels, the evidences that illustrated *O. taihangensis* as a separate species from *O. longilobus* were only leaf form and involucres. So, these two species were once regarded as a species. In this study, the analyses of the combined cpDNA and nrDNA matrix support the monophyly of two clades (Figure 3A, Figure 4A, and Figure S1). The phylogenetic relationships among all populations show an overall congruence with the result of the haplotype network (Figure 3B and Figure 4B). All haplotypes are split into two major groups according to cpDNA and nrDNA data. The populations from two distinct clades approximately exhibited specific geographical distributions in Taihang Mountains. SAMOVA analysis identified two approximately defined groups corresponding to *O. longilobus* and *O. taihangensis* populations at every level of divergence. Within DNA sequences of cpDNA and ITS, the total of thirty polymorphic sites harbored in populations. Unique haplotypes found in *O. longilobus* and *O. taihangensis* populations respectively. Moreover, a significant variation based on AMOVA occurred between *O. longilobus* and *O. taihangensis* (*F*_STcpDNA_ = 0.482 and *F*_STnrDNA_ = 0.466, Table 2). By used SRAP and cpSSR markers [58]–[59], AMOVA indicated that a significant genetic differentiation between *O. longilobus* and *O. taihangensis*. Accordingly, our molecular results support that *O. longilobus* and *O. taihangensis* were regarded as two species rather than a species. That result is consistent with the point of Hu [25]. The divergence times between *O. longilobus* and *O. taihangensis* (Figure 3A) indicate that genetic divergence between two species occurred in early Pleistocene. This split was related to the ecological and geographical habitat changes resulting from climate oscillations during the Quaternary glacial periods and complicated topography with the uplift of Taihang Mountains.

### Geographic patterns of genetic diversity and structure

The high levels of genetic diversity were observed in *O. longilobus* and *O. taihangensis* populations (Table 1). These results are similar to previous research on herbaceous plants in East Asia and significantly exceed the average value of 0.67 for the 170 plant species documented [8], [12], [19], [31], [60]. In *O. longilobus*, the WDS population obtained the most haplotypes and highest genetic diversity, followed by the SHS population. This suggests the presence of two genetic diversity centers for *O. longilobus*, i.e., the regions where WDS and SHS populations are located. For *O. taihangensis*, the BJY and WML populations, which obtained the high genetic diversity, were genetic diversity centers for *O. taihangensis*. Significant genetic divergence and a highly structure were illustrated in all studied populations (Figure 3, Figure 4, and Table 2). For *O. longilobus* (*F*_STcpDNA_ = 0.607, *F*_STnrDNA_ = 0.682) and *O. taihangensis* (*F*_STcpDNA_ = 0.686, *F*_STnrDNA_ = 0.805), the results of our analysis suggested that significant variation was detected from among populations rather than from variation within groups (Table 2). Moreover, very high genetic differentiation in *O. longilobus* and *O. taihangensis* greatly exceeding the mean value (*G*_ST_ = 0.22) reported by Nybom [61].

Species genetic diversity and structure can be affected by life history traits (e.g., life cycle, breeding system, pollination mechanism) and environmental effects (e.g., geographical range, climate, topography) [61]–[62]. *O. longilobus* and *O. taihangensis* possess a sexual reproductive mode and a relatively long life cycle as perennial herbs, all of which are traits associated with low total genetic diversity [63]–[64]. However, high genetic diversity was observed in our study, and this diversity appears to result from high among-population genetic variation. It has been widely documented that the dispersal of pollen and seeds in plant species is strongly linked to the development of a population genetic structure in bi-parentally inherited (nuclear-encoded) and maternally inherited (cytoplasmic) genetic markers [12], [31], [61], [62], [64]. Generally, outcrossing taxa with high seed dispersal capacity retain the majority of both types of genetic marker variation within populations, whereas selfing (and/or asexual) taxa with restricted seed dispersal allocate the majority of such variation among populations. *O. longilobus* and *O. taihangensis* are self-compatible plant species [25]. These two species, therefore, might be expected to have a heterogeneous distribution of both cpDNA and nrDNA variation among populations. Taken at face value, the above results may suggest that restricted gene flow via pollen and seed has resulted in significant population genetic differentiation in *O. longilobus* (*N*_mcpDNA_ = 0.12; *N*_mnrDNA_ = 0.10) and *O. taihangensis* (*N*_mcpDNA_ = 0.19; *N*_mnrDNA_ = 0.14). The physiographic complexity of Taihang Mountains and the deeply carved valleys and ravines between the inhabit areas probably would imposes significant barriers to gene flow among populations of *O. longilobus* and *O. taihangensis*. The genetic divergence between the two lineages occurred at 1.28 Ma, corresponding to the early Pleistocene. This divergence falls within the glaciations during the Taku Glaciation (1.05–1.20 Ma). The glaciers and/or extremely low temperature in the mountains might also have created barriers to gene flow between geographically isolated populations, which therefore promoted the divergence. For both marker systems, we generally observed a good fit of these populations to an IBD model. When coupled with high levels of population subdivision, such conditions strongly suggest that geographical isolation had a larger historical role in extant population structure compared with limited pollen and seed flow alone. The habitats of *O. longilobus* and *O. taihangensis* are either rocky or massifs, and these species grow discontinuously in different habitats along the altitudinal gradient from 700 to 1500 m, in which the variable micro-surrounding, complex topography, and great altitudinal variability of the region might promote the high degree of genetic population differentiation [65]–[66]. Limited migration coupled with a heterogeneous environment could promote local adaptation and fixation of different alleles in *O. longilobus* and *O. taihangensis* populations.

### Phylogeographical structure and inference of demographic history

Both *O. longilobus* and *O. taihangensis* populations have a significant phylogeographic structure. The current distribution of haplotypes could be a result of past climatic changes related to the advance/retreat of glaciations [11]. During the Quaternary periods, climatic oscillations resulted in repeated drastic environmental changes, which further caused massive range shifts of most plants and animals, leading to accumulated genetic differences and particular phylogeographic patterns [2]–[3].

Our analyses suggested that *Opisthopappus* originated during the Pliocene. Before the Pleistocene, the current range of Taihang Mountains would have been covered by grassland with roughly similar geographic conditions [67]. As herbs, *O. longilobus* and *O. taihangensis* can disperse following grassland migration. On this basis, we suspect that *O. longilobus* and *O. taihangensis* were once distributed throughout Taihang Mountains and its adjacent region. With uplift of Taihang Mountains [27] and climate fluctuation of Quaternary glacial periods, *O. longilobus* and *O. taihangensis* were fragmented and forced into the refuge areas. The flora would migrate and expand during interglacial and glacial periods. This migration and expansion resulted in ecological displacement, which might have resulted in populations situated at different spatial-temporal scales. Different habitats might enhance the isolation of plant populations and prevent gene flow, which in turn could lead to incipient allopatric speciation or further allopatric speciation [12], [18], [19], [68], [69]. During the Quaternary periods, the paleovegetation in Taihang Mountains repeatedly appear the replacement of grasslands and forests cycles [67]. Those multiple replacements likely fragmented and isolated the habitat of herbs. Therefore, the refuge areas for *O. longilobus* and *O. taihangensis* would have remained separated and fragmented at different spatial-temporal scales for a long time. Moreover, dramatic geological changes induced by the Taihang Mountains uplift contributed to the extremely complicated landscape, which likely acted as a major driving force for the separation of the *O. longilobus* and *O. taihangensis*. Divergent selection between populations in contrasting environments, longer temporal and spatial separation and fragmentation would accelerate their differentiation, and eventually promote allopatric speciation.

Following this divergence, *O. longilobus* and *O. taihangensis* underwent strikingly different histories. For *O. longilobus*, long-distance dispersal and colonization may be a major historical process. *O. longilobus* was divided into different lineages around 0.89 Ma, which fell within the interglacial stage between the Taku Glaciation and Lushan Glaciation. The paleovegetation grasslands of Taihang Mountains [67] might be regarded as a corridor connecting the currently isolated populations. Populations of *O. longilobus* in Hebei province may have extended across this corridor and reached Henan province. Haplotypes H1 and Hn1 located in populations LLS and WDS or LLS and SBY imply this long dispersal (Table 2). The 10 bp insertions of *ndh*J-*trn*L occurring in population LLS also supported this viewpoint (Table S1). Subsequently, with the advent of inter-glaciations or post-glaciations, the climate changed, which led to the paleovegetation of Taihang Mountains experiencing a transition from grasslands to forests. The corridor for *O. longilobus* migration gradually disappeared, which would have resulted in isolation of the population [11], [12], [68]. Additionally, we did not detect any population expansion due to the clear multimodel mismatch distribution (Figure S2). The complex geological conditions of Taihang Mountains would limit *O. longilobus* population expansion on a macro scale and thus restricting *O. longilobus* to sustain in situ during the Quaternary periods, perhaps by moving upwards and downwards in their mountain ranges. For *O. taihangensis*, a pattern of population expansion was observed, because the observed mismatch distribution for cpDNA and nrDNA haplotypes was unimodal (a pattern consistent with expansion) (Figure S2). This expansion was dated to be 0.78 Ma, which corresponding the interglacial stage between the Taku Glaciation and Lushan Glaciation. The above results suggest an expansion consistent with a range expansion and re-colonization before the mainly uplift of Taihang Mountains. With the uplift of Taihang Mountains, large geographical distances and significant topographical barriers could restrict gene flow and limit any large-scale population expansion [12], [70]. The cpDNA sequences characteristic of the *trn*L-*trn*F and *ndh*J-*trn*L intergenic spacers further confirmed this hypothesis (Table S1).

Crandall and Templeton [71] suggest that ancestral haplotypes are located in a central position within the haplotype phylogeographical network and that potential refugia are usually characterized by widely dispersed ancestral haplotypes and other tip and unique haplotypes. Geographic areas displaying increased levels of genetic diversity are thus good candidates in the search for past refuges [8], [34]. These regions are characterized by relatively stable ecological conditions during environmental fluctuations, which foster the accumulation of genetic diversity [72]. Most *O. longilobus* individuals or populations gathered in lineage I (Figure 3 and Figure 4). Haplotypes H3 and Hn8 are located in the center of the haplotype network. And Haplotype H3 is detected in three populations (WDS, SHS and WML), Hn8 in two populations (WDS and SHS) (Figure 2). WDS and SHS are located in the southern part of Hebei province and have higher haplotype diversity (Table 1). Thus, we suggest that the WDS and SHS populations' regions were two major potential refugia for *O. taihangensis*. These regions may therefore have been characterized by a relatively mild climate, potentially containing microclimatic environments able to impart relative stability to a range of habitats. Lineage II consisted of all populations of *O. taihangensis* and some individuals of *O. longilobus*. Haplotypes H1 and Hn1, which are located in the center of the haplotype network, were the most dominant haplotypes found in five and ten populations, respectively. Moreover, some populations in these haplotypes (H1 and Hn1) had higher haplotype diversity (Table 1, e.g., BJY, WML, and YTS). Thus, we suggest that there were multiple small refuges located in Shanxi and Henan provinces during the last glacial maximum or earlier cold periods for *O. taihangensis*. The above populations of *O. taihangensis*, distributed in southern Taihang Mountains, would have been provided with a complex and stable environment. Thus, the potential refugia area may have been located in those regions.

However, our comprehensive sampling showed that assessing the species-level relationships in our study is complicated by the fact that not all cpDNA and nrDNA haplotypes are species-specific. Incongruence between the species boundaries and the genealogy of their cpDNA and nrDNA sequences is illustrated by the sharing of haplotypes (e.g., H1 and Hn8) between the two species. On the other hand, several haplotypes were found within a single species (Table 1 and Figure 2). Haplotype distribution does not strictly follow species circumscription. Moreover, an association of haplotypes with geographically circumscribed regions rather than with taxonomic boundaries is a phenomenon observed in *O. longilobus* and *O. taihangensis*. The sharing of haplotypes and lack of reciprocal monophyly might be explained by the persistence of ancestral polymorphisms during speciation events and/or exchange of genes by inter-specific hybridization (Table S1 and Figure S1). These two species have similar habitats and the same period of flowering [25], and possible visitation by the same pollinators in the absence of physiological or genetic reproductive barriers might have enabled hybridization events. Nonetheless, further work on chromosomes and the sequencing of additional genomic regions are needed to make more in-depth conclusions about the hybrid status of these taxa.

## Conclusions

In summary, *O. longilobus* and *O. taihangensis* have a high level of genetic diversity. The following phylogeographical structure and historical scenario of *O. longilobus* and *O. taihangensis* can also be drawn. The vicariance following the uplift of Taihang Mountains and a transition of paleovegetation of Taihang Mountains mainly shaped the present distribution of these two species. The above findings imply that multiple small refugia could have been maintained in the Taihang Mountains regions, which allowed *O. longilobus* and *O. taihangensis* to persist in situ and maintain sizable haplotype and nucleotide diversity at the lineage-wide scale during the glaciations. The current distribution pattern of *O. longilobus* and *O. taihangensis* refuges is similar to that of other East Asian plants (e.g., *Cathaya argyrophylla*, *Picea crassifolia*, *Sinopodophyllum hexandrum*, *Lagochilus Bunge* ex Bentham) [8], [20], [73], with multiple refuges sustained across their distribution ranges. Furthermore, complex topography rendered these refuge areas both isolated and fragmented in different geographic units. Geographical and ecological isolation likely restricted seed and pollen migration ability. The isolation and fragmentation further promoted the intra-specific split. These results support the conclusion that the high plant diversity and endemism found in central/northern China and throughout eastern Asia has mainly resulted from allopatric speciation due to the complex topography of Mountains and allopatric fragmentation during the late Tertiary and Quaternary periods.

## Supporting Information

Figure S1 **Phylogenetic analyses based on ITS sequences.**
(DOC)

Figure S2 **Pairwise mismatch distribution analyses (MDAs) for** ***O. taihangensis*** **(A, B) and** ***O. longilobus*** **(C, D) populations inferred from cpDNA and ITS sequences.**
(DOC)

Table S1 **The cpDNA sequences characteristic of** ***trn*** **L-** ***trn*** **F and** ***ndh*** **J-** ***trn*** **L intergenic spacers.**
(DOC)

## References

[1] Avise JC, Walker D, Johns GC (1998) Speciation durations and Pleistocene effects on vertebrate phylogeography. Proceedings of the Royal Society of London Series B Biological Sciences 265: 1707–1712. 9787467 10.1098/rspb.1998.0492 PMC1689361

[2] Hewitt GM (2000) The genetic legacy of the Quaternary ice ages. Nature 405: 907–913. 10879524 10.1038/35016000

[3] Hewitt GM (2004) Genetic consequences of climatic oscillations in the Quaternary. Philosophical Transactions of the Royal Society of London Series B Biological Sciences 359: 183–195. 15101575 10.1098/rstb.2003.1388 PMC1693318

[4] Willis KJ, Whittaker RJ (2002) Species diversity-scale matters. Science 1245–1248. 10.1126/science.1067335 11847328

[5] Turchetto-Zolet AC, Cruz F, Vendramin GG, Simon MF, Salgueiro F, et al (2012) Large-scale phylogeography of the disjunct Neotropical tree species *Schizolobium parahyba* (Fabaceae-Caesalpinioideae). Molecular Phylogenetics and Evolution 65: 174–182. 22750114 10.1016/j.ympev.2012.06.012

[6] Xu JW, Chu KH (2012) Genome scan of the mitten crab *Eriocheir sensu stricto* in East Asia: Population differentiation, hybridization and adaptive speciation. Molecular Phylogenetics and Evolution 64: 118–129. 22465485 10.1016/j.ympev.2012.03.009

[7] Cao MM, Jin YT, Liu NF, Ji WH (2012) Effects of the Qinghai–Tibetan Plateau uplift and environmental changes on phylogeographic structure of the *Daurian Partridge* (Perdix dauuricae) in China. Molecular Phylogenetics and Evolution 65: 823–830. 22940153 10.1016/j.ympev.2012.08.004

[8] Meng HH, Zhang ML (2013) Diversification of plant species in arid Northwest China: Species-level phylogeographical history of *Lagochilus Bunge* ex Bentham (Lamiaceae). Molecular Phylogenetics and Evolution 68: 398–409. 23629053 10.1016/j.ympev.2013.04.012

[9] Avise JC (2000) Phylogeography: The History and Formation of Species. Harvard University Press, Cambridge.

[10] Yu G, Chen X, Ni J, Cheddadi R, Guiot J, et al (2000) Palaeovegetation of China: a pollen databased synthesis for the mid-Holocene and last glacial maximum. Journal of Biogeography 27: 635–664.

[11] Zhang YH, Volis S, Sun H (2010) Chloroplast phylogeny and phylogeography of *Stellera chamaejasme* on the Qinghai-Tibet Plateau and in adjacent regions. Molecular Phylogenetics and Evolution 57: 1162–1172 20828627 10.1016/j.ympev.2010.08.033

[12] Zhao YP, Qi ZC, Ma WW, Dai QY, Li P, et al (2013) Comparative phylogeography of the *Smilax hispida group* (Smilacaceae) in eastern Asia and North America – Implications for allopatric speciation, causes of diversity disparity, and origins of temperate elements in Mexico. Molecular Phylogenetics and Evolution 68: 300–311. 23578597 10.1016/j.ympev.2013.03.025

[13] Xie KQ, Zhang ML (2013) The effect of Quaternary climatic oscillations on *Ribes meyeri* (Saxifragaceae) in northwestern China. Biochemical Systematics and Ecology 50: 39–47.

[14] Tian B, Liu RR, Wang LY, Qiu Q, Chen KM, et al (2009) Phylogeographic analyses suggest that a deciduous species (Ostryopsis davidiana Decne., Betulaceae) survived in northern China during the Last Glacial Maximum. Journal of Biogeography 36: 2148–2155.

[15] Jia DR, Liu TL, Wang LY, Zhou DW, Liu JQ (2011) Evolutionary history of an alpine shrub *Hippophaë tibetana* (Elaeagnaceae): allopatric divergence and regional expansion. Biological Journal of Linnean Society 102: 37–50.

[16] Chen KM, Abbott RJ, Milne RI, Tian XM, Liu JQ (2008) Phylogeography of *Pinus tabulaeformis* Carr. (Pinaceae), a dominant species of coniferous forest in northern China. Molecular Ecology 17: 4276–4288. 19378405 10.1111/j.1365-294x.2008.03911.x

[17] Bai WN, Liao WJ, Zhang DY (2010) Nuclear and chloroplast DNA phylogeography reveal two refuge areas with asymmetrical gene flow in a temperate walnut tree from East Asia. New Phytologist 188: 892–901. 20723077 10.1111/j.1469-8137.2010.03407.x

[18] Harrison SP, Yu G, Takahara H, Prentice IC (2001) Palaeovegetation: diversity of temperate plants in East Asia. Nature 413: 129–130. 11557970 10.1038/35093166

[19] Qiu YX, Guan BC, Fu CX, Comes HP (2009) Did glacials and/or interglacials promote allopatric incipient speciation in East Asian temperate plants? Phylogeographic and coalescent analyses on refugial isolation and divergence in *Dysosma versipellis* . Molecular Phylogenetics and Evolution 51: 281–293. 19405195 10.1016/j.ympev.2009.01.016

[20] Li Y, Stocks M, Hemmilä S, Källman T, Zhu HT, et al (2010) Demographic histories of four spruce (Picea) species of the Qinghai-Tibetan Plateau and neighboring areas inferred from multiple nuclear loci. Molecular Biology and Evolution 27: 1001–1014. 20031927 10.1093/molbev/msp301

[21] Wu CY (1995) Vegetation of China. Science Press, Beijing.

[22] Zhao SL, Li GG (1990) Desertization on the shelves adjacent China in the Later Pleistocene. Oceanologia et Limnologia Sinica 8: 289–298.

[23] Liu KB (1988) Quaternary history of the temperate forests of China. Quaternary Sciences Reviews 7: 1–20.

[24] Zhu LM (2008) Spider community structure in fragmented habitats of Taihang Mountain area, China. Thesis for Master' Degree, Hebei Univiersity.

[25] Hu X (2008) Preliminary studies on inter-generic hybridization within *Chrysanthemum* in broad sense (III). Thesis for Master' Degree, Beijing Forestry University.

[26] Gong MQ (2010) Uplifting process of southern Taihang Mountain in Cenozoic. Chinese Academy of Geological Science Thesis for Doctor' Degree.

[27] Wu C, Zhang XQ, Ma YH (1999) The Taihang and Yan Mountains rose mainly in Quarteranary. Norht China Earthquake Sciences 17 (3) 1–7.

[28] Gao LM, Möller M, Zhang XM, Hollingsworth ML, Liu J, et al (2007) High variation and strong phylogeographic pattern among cpDNA haplotypes in *Taxus wallichiana* (Taxaceae) in China and North Vietnam. Molecular Ecology 16: 4684–4698. 17908214 10.1111/j.1365-294X.2007.03537.x

[29] Zhou TH, Li S, Qian ZQ, Su HL, Huang ZH, et al (2010) Strong phylogeographic pattern of cpDNA variation reveals multiple glacial refugia for *Saruma henryi* Oliv. (Aristolochiaceae), an endangered herb endemic to China. Molecular Phylogenetics and Evolution 57: 176–188. 20637294 10.1016/j.ympev.2010.07.001

[30] Wolfe KH, Li WH, Sharp PM (1987) Rates of nucleotide substitution vary greatly among plant mitochondria, chloroplast, and nuclear DNAs. Proceeding of the National Academy of Sciences of the United States of America 84: 9054–9058. 10.1073/pnas.84.24.9054 PMC299690 3480529

[31] Petit RJ, Duminil J, Fineschi S, Salvini D, Vendramin GG (2005) Comparative organization of chloroplast, mitochondrial and nuclear diversity in plant populations. Molecular Ecology 14: 689–701. 15723661 10.1111/j.1365-294X.2004.02410.x

[32] Zhang RY, Song G, Qu YH, Alström P, Ramos R, et al (2012) Comparative phylogeography of two widespread magpies: Importance of habitat preference and breeding behavior on genetic structure in China. Molecular Phylogenetics and Evolution 65: 562–572. 22842292 10.1016/j.ympev.2012.07.011

[33] Zhang Q, Chiang TY, George M, Liu JQ, Abbott RJ (2005) Phylogeography of the Qinghai-Tibetan Plateau endemic *Juniperus przewalskii* (Cupressaceae) inferred from chloroplast DNA sequence variation. Molecular Ecology 14: 3513–3524. 16156819 10.1111/j.1365-294X.2005.02677.x

[34] Zhao C, Wang CB, Ma XG, Liang QL, He XJ (2013) Phylogeographic analysis of a temperate-deciduous forest restricted plant (*Bupleurum longiradiatum* Turcz.) reveals two refuge areas in China with subsequent refugial isolation promoting speciation. Molecular Phylogenetics and Evolution 68: 628–643. 23624194 10.1016/j.ympev.2013.04.007

[35] Wang FZ, Tang J, Chen XQ, Liang SJ, Dai LK, et al.. (1978) Liliaceae. In: Editorial Board of the Flora of China of the China Science Academy. Flora of China 73–74 p.

[36] Ding BZ, Wang SY (1998) Flora of Henan. Henan Science & Technology Press, Zhengzhou.

[37] Doyle JJ, Doyle JL (1987) A rapid DNA isolation procedure for small quantities of fresh leaf material. Phytochemistry Bulletin 19: 11–15.

[38] Taberlet PT, Gielly L, Patou G, Bouvet J (1991) Universal primers for amplification of three noncoding regions of chloroplast DNA. Plant Molecular Biology 17: 1105–1109. 1932684 10.1007/BF00037152

[39] Vijverberg K, Bachmann K (1999) Molecular evolution of a tandemly repeated trnF (GAA) gene in the chloroplast genomes of *Microseris* (Asteraceae) and the use of structural mutations in phylogenetic analyses. Molecular Biology and Evolution 16 (10) 1329–1340. 10563014 10.1093/oxfordjournals.molbev.a026043

[40] White TJ, Bruns T, Lee S, Taylor J (1990) Amplification and direct sequencing of fungal ribosomal RNA genes for phylogenetics. In: Innis MA, Gelfand DH, Shinsky JJ, White TJ (eds), PCR Protocols: A Guide to Methods and Applications. Academic Press, San Diego 315–322 p.

[41] Thompson JD, Gibson TJ, Plewniak F, Jeanmougin F, Higgins DG (1997) The ClustalX windows interface: flexible strategies for multiple sequence alignment aided by quality analysis tools. Nucleic Acids Research 25: 4876–4882. 9396791 10.1093/nar/25.24.4876 PMC147148

[42] Rozas J, Sanchez-DelBarrio JC, Messeguer X, Rozas R (2003) DnaSP, DNA polymorphism analyses by the coalescent and other methods. Bioinformatics 19: 2496–2497. 14668244 10.1093/bioinformatics/btg359

[43] Pons O, Petit RJ (1996) Measuring and testing genetic differentiation with ordered versus unordered alleles. Genetics 144: 1237–1245. 8913764 10.1093/genetics/144.3.1237 PMC1207615

[44] Excoffier L, Laval G, Schneider S (2005) Arlequin ver. 3.0: an integrated software package for population genetics data analysis. Evolutionary Bioinformatics Online 1: 47–50. PMC2658868 19325852

[45] Dupanloup I, Schneider S, Excoffier L (2002) A simulated annealing approach to define the genetic structure of populations. Molecular Ecology 11: 2571–2581. 12453240 10.1046/j.1365-294x.2002.01650.x

[46] Polzin T, Daneshmand SV (2003) On Steiner trees and minimum spanning trees in hypergraphs. Operations Research Letters 31: 12–20.

[47] Clement M, Posada D, Crandall KM (2000) TCS: a computer program to estimate gene genealogies. Molecular Ecology 9: 1657–1660. 11050560 10.1046/j.1365-294x.2000.01020.x

[48] Drummond AJ, Rambaut A (2007) BEAST: Bayesian evolutionary analysis by sampling trees. BMC Evolutionary Biology 7: 214. 17996036 10.1186/1471-2148-7-214 PMC2247476

[49] Heled J, Drummond AJ (2010) Bayesian inference of species trees from multilocus data. Molecular Biology and Evolution 27: 570–580. 19906793 10.1093/molbev/msp274 PMC2822290

[50] Posada D (2008) JModelTest: phylogenetic model averaging. Molecular Biology and Evolution 25: 1253–1256. 18397919 10.1093/molbev/msn083

[51] Ronquist F, Huelsenbeck JP (2003) MrBayes 3: Bayesian phylogenetic inference under mixed models. Bioinformatics 19: 1572–1574. 12912839 10.1093/bioinformatics/btg180

[52] Rambaut A, Drummond AJ (2009) Tracer v1.5. <http://tree.bio.ed.ac.uk/software/tracer/>.

[53] Graur D, Li WH (200) Fundamentals of Molecular Evolution, second ed., Sinauer Associates Inc., Sunderland, Massachusetts.

[54] Tajima F (1989) Statistical method for testing the neutral mutation hypothesis by DNA polymorphism. Genetics 123: 585–595. 2513255 10.1093/genetics/123.3.585 PMC1203831

[55] Fu YX (1997) Statistical tests of neutrality of mutations against population growth, hitchhiking and background selection. Genetics 147: 915–925. 9335623 10.1093/genetics/147.2.915 PMC1208208

[56] Harpending HC (1994) Signature of ancient population growth in a low-resolution mitochondrial DNA mismatch distribution. Human Biology 66: 591–600. 8088750

[57] Jensen JL, Bohonak AJ, Kelley ST (2005) Isolation by Distance, Web Service. BMC Genet. 6, 13 (Version 3.16, <http://ibdws.sdsu.edu/>). 10.1186/1471-2156-6-13 PMC1079815 15760479

[58] Wang YL (2013) Chloroplast microsatellite diversity of *Opisthopappus* Shih. Plant Systematic and Evolution 299: 1849–1858.

[59] Wang YL, Yan GQ (2013) Genetic diversity and population structure of *Opisthopappus longilobus* and *Opisthopappus taihangensis* (Asteraceae) in China determined using sequence related amplified polymorphism markers. Biochemical Systematics and Ecology 49: 115–124.

[60] Wang YL, Li X, Guo J, Guo ZG, Li SF, et al (2010) Chloroplast DNA phylogeography of *Clintonia udensis* Trautv. & Mey. (Liliaceae) in East Asia. Molecular Phylogenetics and Evolution 55: 721–732. 20172032 10.1016/j.ympev.2010.02.010

[61] Nybom H (2004) Comparison of different nuclear DNA markers for estimating intraspecific genetic diversity in plants. Molecular Ecology 13: 1143–1155. 15078452 10.1111/j.1365-294X.2004.02141.x

[62] Loveless MD, Hamrick JL (1984) Ecological determinants of genetic structure in plant populations. Annual Review of Ecology and Systematics 15: 65–95.

[63] Hamrick JL, Godt MJW (1989) Allozyme diversity in plant species. In: Brown AHD, Clegg MT, Kahler AL, Weir BS (eds.), Plant Population Genetics. Breeding and Genetic Resources. Sinauer, Sunderland MA 43–63 p.

[64] Hamrick JL, Godt MJW (1996) Effects of life history traits on genetic diversity in plant species. Philosophical Transactions of the Royal Society of London Series B Biological Sciences 351: 1291–1298.

[65] Till-Bottraund I, Gaudeul M (2002) Intraspecific genetic diversity in alpine plants. In: Körner C, Spehn EM (eds.), Mountain Biodiversity: A Global Assessment. Parthenon Publishing, New York 23–34 p.

[66] Liu L, Hao ZZ, Liu YY, Wei XX, Cun YZ, et al (2014) Phylogeography of *Pinus armandii* and its relatives: heterogeneous contributions of geography and climate changes to the genetic differentiation and diversification of Chinese white pines. PLoS one 9 (1) e85920. 24465789 10.1371/journal.pone.0085920 PMC3897548

[67] Yang XL, Xu QH, Zhao HP (1999) The vegetation succession of Taihang Mountains during the late glaciations. Geography and Territorial Research 15 (1) 81–88.

[68] Qiu YX, Fu CX, Comes HP (2011) Plant molecular phylogeography in China and adjacent regions: tracing the genetic imprints of Quaternary climate and environmental change in the world's most diverse temperate flora. Molecular Phylogenetics and Evolution 59: 225–244. 21292014 10.1016/j.ympev.2011.01.012

[69] Qian H, Ricklefs RE (2001) Diversity of temperate plants in East Asia–reply. Nature 413: 130. 10.1038/35093166 11557970

[70] Chou YW, Thomas PI, Ge XJ, LePage BA, Wang CN (2011) Refugia phylogeography of Taiwaniain East Asia. Journal of Biogeography 38: 1992–2005.

[71] Crandall KA, Templeton AR (1993) Empirical tests of some predictions from coalescent theory with applications to intraspecific phylogeny reconstruction. Genetics 134: 959–969. 8349118 10.1093/genetics/134.3.959 PMC1205530

[72] Gong W, Chen C, Dobeš C, Fu CX, Koch MA (2008) Phylogeography of a living fossil: Pleistocene glaciations forced *Ginkgo biloba* L. (Ginkgoaceae) into two refuge areas in China with limited subsequent postglacial expansion. Molecular Phylogenetics and Evolution 48: 1094–1105. 18554931 10.1016/j.ympev.2008.05.003

[73] Wang HW, Ge S (2006) Phylogeography of the endangered *Cathaya argyrophylla* (Pinaceae) inferred from sequence variation of mitochondrial and nuclear DNA. Molecular Ecology 15: 4109–4122. 17054506 10.1111/j.1365-294X.2006.03086.x

