# Supplementary figures and images for: Molecular Phylogeography and Population Genetic Structure of O. longilobus and O. taihangensis (Opisthopappus) on the Taihang Mountains
Source: PLoS One. 2014 Aug 22;9(8):e104773. doi: 10.1371/journal.pone.0104773 (PMC4141751; doi:10.1371/journal.pone.0104773)

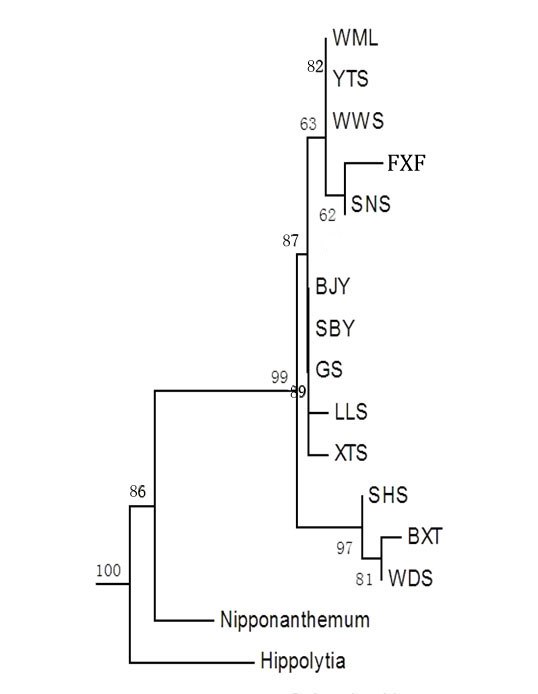


**Figure S1. The phylogenetic analyses based on ITS sequences**

Supplement: Figure S1 — Phylogenetic analyses based on ITS sequences. (DOC) [file pone.0104773.s001.doc]
