# Supplementary material for: Molecular Phylogeography and Population Genetic Structure of O. longilobus and O. taihangensis (Opisthopappus) on the Taihang Mountains
Source: PLoS One. 2014 Aug 22;9(8):e104773. doi: 10.1371/journal.pone.0104773 (PMC4141751; doi:10.1371/journal.pone.0104773)
Supplement: Figure S2 — Pairwise mismatch distribution analyses (MDAs) for O. taihangensis (A, B) and O. longilobus (C, D) populations inferred from cpDNA and ITS sequences. (DOC) [file pone.0104773.s002.doc]

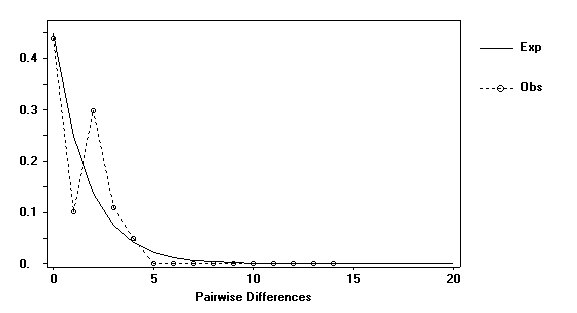

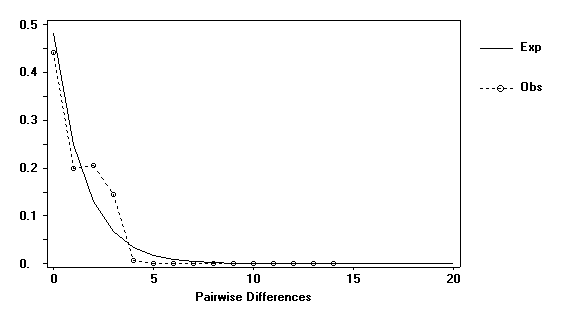


1. (B)


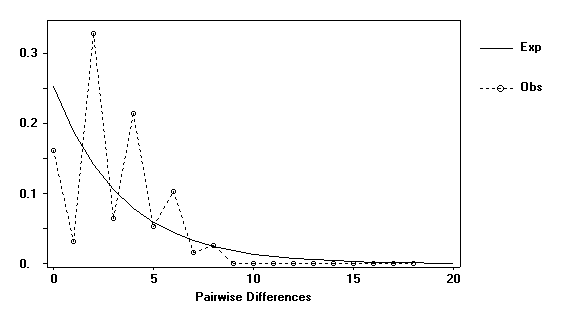

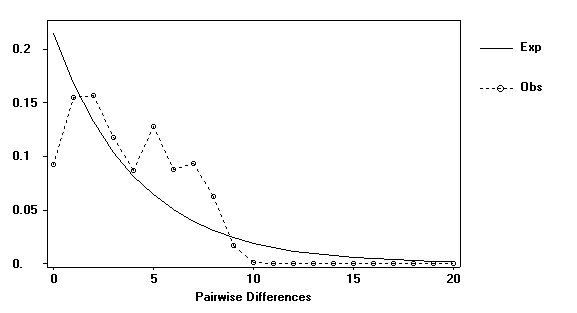


(C) (D)

**Figure S2. Pair-wise mismatch distribution analyses (MDAs) for** ***O. taihangensis* (A, B) and *O. longilobus* (C, D) population inferred from cpDNA and ITS sequences**.
