# Supplementary material for: Molecular Phylogeography and Population Genetic Structure of O. longilobus and O. taihangensis (Opisthopappus) on the Taihang Mountains
Source: PLoS One. 2014 Aug 22;9(8):e104773. doi: 10.1371/journal.pone.0104773 (PMC4141751; doi:10.1371/journal.pone.0104773)
Supplement: Table S1 — The cpDNA sequences characteristic of trn L- trn F and ndh J- trn L intergenic spacers. (DOC) [file pone.0104773.s003.doc]

**Table S1.** The cpDNA sequrences characteristic of *trn*L-*trn*F and *ndh*J-*trn*L intergenic spacers

|  | *trn*L-*trn*F | | | | | | | | | | *ndh*J-*trn*L | | | | |
| --- | --- | --- | --- | --- | --- | --- | --- | --- | --- | --- | --- | --- | --- | --- | --- |
|  | 65 | 238 | 588-591 | 607 | 639 | 743 | 748 | 757 | 784 | 791-792 | 1061 | 1063 | 1108 | 1264-1273 | 1697 |
| LLS | A | A | ---- | T | C | G | C | T | C | TT | A | A | G | TTGTATGTAT | A |
| SBY | A | A | ---- | T | C | G | C | T | C | TT | - | C | G | ---------- | C |
| WDS1 | A | A | GATT | C | C | G | G | T | G | GG | A | A | G | TTGTATGTAT | A |
| WDS2 | A | A | GATT | C | C | G | C | T | C | TT | A | A | G | TTGTATGTAT | A |
| WDS3 | A | A | GATT | C | C | G | G | T | G | GG | A | A | G | TTGTATGTAT | A |
| WDS4 | A | A | GATT | T | T | G | C | T | C | TT | A | A | G | TTGTATGTAT | A |
| WDS5 | A | A | GATT | T | C | G | C | T | C | TT | A | A | G | TTGTATGTAT | A |
| WDS6 | A | A | GATT | T | C | C | C | G | C | TT | A | A | G | TTGTATGTAT | A |
| WDS7 | T | C | GATT | T | C | G | C | T | C | TT | A | A | G | TTGTATGTAT | A |
| BXT | A | C | GATT | C | C | G | C | T | C | TT | - | C | G | ---------- | C |
| SHS1 | A | C | GATT | C | C | G | G | T | G | GG | A | A | G | TTGTATGTAT | A |
| SHS | A | C | GATT | C | C | G | C | T | C | TT | A | A | G | TTGTATGTAT | A |
| GS | A | A | ---- | T | C | G | C | T | C | TT | A | A | G | TTGTATGTAT | A |
| YTS1 | A | A | ---- | T | C | G | C | T | C | TT | - | C | G | --------- | C |
| YTS2 | A | A | ---- | T | C | G | C | T | C | TT | - | A | A | TTGTATGTAT | A |
| XT1 | A | A | ---- | T | C | G | C | T | C | TT | - | A | A | TTGTATGTAT | A |
| XT2 | A | A | ---- | T | C | G | C | T | C | TT | - | C | G | --------- | C |
| SNS | A | A | ---- | T | C | G | C | T | C | TT | - | C | G | --------- | C |
| FXF | A | A | ---- | T | C | G | C | T | C | TT | - | C | G | --------- | C |
| WWS | A | A | ---- | T | C | G | C | T | C | TT | - | C | G | --------- | C |
| WML1 | A | A | ---- | T | C | G | C | T | C | TT | - | C | G | TTGTATGTAT | A |
| WML2 | A | C | GATT | C | C | G | C | T | C | TT | - | C | G | TTGTATGTAT | A |
| BJY1 | A | A | ---- | T | C | G | C | T | C | TT | - | C | G | --------- | A |
| BJY2 | A | A | ---- | T | C | G | C | T | C | TT | A | A | G | TTGTATGTAT | A |
